# Effect of Low‐Dose Propofol Administered at the End of Sevoflurane Anesthesia on Emergence Agitation in Pediatric Patients Undergoing MRI: A Randomized Controlled Trial

**DOI:** 10.1155/anrp/6532790

**Published:** 2026-09-01

**Authors:** Thitinuch Ruenhunsa, Santhita Pimonbut, Peerapong Sangsungnern, Prathana Wittayapairoch, Darunee Sripadungkul, Narumon Wongsaneha, Wanatnarin Sorit, Palardej Narrutto

**Affiliations:** ^1^ Department of Anesthesiology, Faculty of Medicine, Khon Kaen University, Khon Kaen, Thailand, kku.ac.th; ^2^ Division of Nursing, Srinagarind Hospital, Faculty of Medicine, Khon Kaen University, Khon Kaen, Thailand, kku.ac.th

**Keywords:** children, delirium, inhalational anesthesia, nonoperating room anesthesia

## Abstract

**Background:**

Emergence agitation (EA) is a common postanesthetic problem in pediatric patients, particularly with sevoflurane anesthesia, and may lead to injury to patients, prolonged postanesthesia care unit (PACU) stay, and a risk of long‐term maladaptive behavioral changes. Evidence regarding EA in nonpainful procedures such as magnetic resonance imaging (MRI) remains inconclusive. This study evaluated the effect of low‐dose propofol at the end of sevoflurane anesthesia on EA in pediatric patients undergoing MRI.

**Methods:**

In this randomized controlled trial, 130 pediatric patients aged 2–8 years undergoing MRI under sevoflurane anesthesia with a laryngeal mask airway were enrolled. Patients were randomly assigned to receive either Group P (*n* = 61), receiving intravenous propofol 0.5 mg/kg, or Group N (*n* = 61), receiving normal saline at the end of anesthesia. EA was assessed using the Pediatric Anesthesia Emergence Delirium (PAED) scale, with EA defined as a score > 12 within the first 30 min of PACU stay. Primary analysis was performed on a per‐protocol approach (*n* = 122), alongside a sensitivity analysis using the intention‐to‐treat approach (*n* = 130). Secondary outcomes included emergence time, PACU stay, adverse events, and requirement for EA rescue medication.

**Results:**

In the per‐protocol analysis (*n* = 122), the incidence of EA was significantly lower in Group P compared with Group N (18.0% vs 39.3%; *p* = 0.01), with a relative risk of 0.46 (95% CI 0.25–0.85). Emergence time, PACU stay, adverse events, and requirement of EA rescue medication were comparable between the two groups.

**Conclusions:**

Administration of propofol at a dose of 0.5 mg/kg at the end of sevoflurane anesthesia significantly reduces EA in pediatric patients undergoing MRI without prolonging recovery or increasing adverse events. Further large‐scale, multicenter studies are warranted to confirm efficacy and evaluate safety.

**Trial Registration:** ClinicalTrials.gov identifier: NCT06218680

## 1. Introduction

Emergence agitation (EA) commonly occurs as a postanesthetic issue in pediatric patients, with 42% of pediatric anesthesiologists perceiving it as a significant concern [1]. It is defined as a dissociation of consciousness during recovery from general anesthesia, manifested as inconsolable crying, restlessness, involuntary physical activity, and unawareness of the environment [2]. The clinical consequences of EA are typically short‐lived and self‐limited; however, it may lead to dislodgement of intravenous catheters, injury to patients or staff, parental dissatisfaction, prolonged postanesthesia care unit (PACU) stay, and a risk of long‐term maladaptive behavioral changes [3, 4]. The incidence of EA has been reported to vary widely, ranging from 10% to 80%, depending on the definitions and diagnostic criteria used, including the Pediatric Anesthesia Emergence Delirium (PAED), Cravero, and Watcha scales [1, 5–7]. Identified risk factors include age between 2 and 6 years; sevoflurane anesthesia; preoperative anxiety; ear, nose, and throat surgery; and postoperative pain [4, 7, 8].

The mechanism of EA in pediatric patients receiving volatile anesthetic agents remains incompletely understood. Low blood solubility volatile anesthetic agents, such as sevoflurane and desflurane, allow for rapid emergence; however, this may result in differential recovery, in which sensory and motor functions recover earlier than cognitive function, thereby increasing the risk of EA [4, 9]. In addition, low concentrations of sevoflurane during emergence have been suggested to induce excitatory neural activity [10].

Propofol is a rapid‐onset, short‐duration intravenous anesthetic commonly used for sedation as well as induction and maintenance of anesthesia. Through its GABAergic effects, propofol facilitates a smooth and gradual emergence while counteracting sevoflurane‐induced excitatory activity during emergence [11]. Given this pharmacological profile, administration of propofol at the end of sevoflurane anesthesia may be a suitable option to reduce EA in pediatric patients. Several studies have demonstrated that intravenous propofol at a dose of 1–3 mg/kg, administered at the end of anesthesia, significantly reduces the incidence of EA in pediatric patients undergoing elective surgery with general anesthesia; however, this strategy may be associated with prolonged recovery time and an increased risk of apnea and airway compromise [11–13]. High doses of propofol are also associated with cardiovascular adverse effects, such as bradycardia and hypotension [14]. Various pharmacological agents, including fentanyl, midazolam, dexmedetomidine, and ketamine, have also been investigated to reduce EA in pediatric anesthesia; nevertheless, no standard practice has been established [3–5, 15, 16].

Magnetic resonance imaging (MRI) in pediatric patients often requires sedation or general anesthesia to ensure immobility and optimal image quality. Sevoflurane anesthesia with a laryngeal mask airway (LMA) is commonly used for pediatric MRI procedures due to its ability to provide adequate depth of anesthesia while maintaining airway patency, particularly during long‐duration scans [17]. Although MRI is a nonpainful procedure, we hypothesized that pediatric patients undergoing MRI may still be at risk for EA associated with sevoflurane anesthesia [18]. Prior MRI‐specific studies on the prevention of EA have evaluated dexmedetomidine or propofol total intravenous anesthesia for maintenance; however, adjunctive low‐dose propofol administered at the end of sevoflurane anesthesia has not been studied in this nonpainful setting [19–21]. A previous study demonstrated that intravenous propofol at a dose of 0.5 mg/kg, administered at the end of anesthesia, significantly reduced the incidence of EA in pediatric patients undergoing elective surgery compared with placebo [10]. Accordingly, in the present study, we selected a low dose of propofol, which is defined as 0.5 mg/kg. This study aimed to determine whether intravenous propofol at a dose of 0.5 mg/kg, administered at the end of anesthesia, reduced the incidence of EA in pediatric patients undergoing MRI under sevoflurane anesthesia.

## 2. Methods

This study was a randomized controlled trial conducted at Srinagarind Hospital, Faculty of Medicine, Khon Kaen University, during January 2024 and December 2025. This study was approved by the Khon Kaen University Ethics Committee in Human Research in accordance with the Declaration of Helsinki, the ICH good clinical guideline (HE661348) on August 8, 2023, and was registered at ClinicalTrials.gov on January 20, 2024. Written informed consent was obtained from the parents or legal guardians of the pediatric patients prior to enrollment.

We enrolled pediatric patients scheduled for MRI procedure under sevoflurane anesthesia using an LMA, aged 2–8 years, and classified as American Society of Anesthesiologists physical status (ASA PS) 1–2. Excluded patients were those with delayed development, psychiatric disorders, neurological disorders, cardiovascular or pulmonary disease, a history of allergy to study drugs, or a history of allergy to egg or soybean. Patients with failed LMA insertion or those who received additional intravenous anesthetic agents during the procedure were withdrawn from the study.

Pediatric patients and their parents were evaluated preoperatively by the attending anesthesiologist. This was conducted either in the admission ward on the evening prior to the procedure for inpatients or in the preoperative area on the morning of procedure for outpatients. Following agreement to participate and provision of written informed consent by the parents or legal guardians, patients were randomly allocated into one of the two study groups using block of four randomization with a computer‐generated random sequence (https://www.randomizer.org/). Patients in Group P received intravenous propofol at a dose of 0.5 mg/kg at the end of anesthesia, whereas those in Group N received 2 mL of normal saline. The sequential randomization codes were enclosed in sealed, opaque envelopes to ensure allocation of concealment. A research team member opened each envelope prior to the induction of anesthesia to reveal the patient’s group assignment and informed the attending anesthesiologist. The study drug was prepared by a research team member and administered by attending anesthesiologist. Due to the distinct visual properties of the study solution—propofol being milky white emulsion and normal saline being transparent—blinding of the attending anesthesiologist was not feasible.

EA in pediatric patients was assessed using the PAED scale, a validated instrument for diagnosing EA. This observational scale consists of five behavioral items: eye contact, purposeful actions, awareness of surroundings, restlessness, and inconsolability. Each item is scored from 0 to 4, with total PAED scores ranging from 0 to 20. A PAED score greater than 12 indicates EA, with a sensitivity of 100% and a specificity of 95% [22].

On the day of the MRI procedure, no sedative premedication was administered to pediatric patients. All patients were transferred to the induction area situated in the magnet‐free safety zone of the MRI facility. In this area, standards of anesthesia monitoring were applied. Parents or legal guardians were allowed to be present in the induction area until the patient was anesthetized. Inhalational induction of anesthesia was performed using 8% sevoflurane administered in 6 L/min of oxygen via a facemask. After achieving of adequate depth of anesthesia, a peripheral intravenous line was placed and appropriately sized LMA was inserted. Throughout the MRI procedure, anesthesia was maintained with a target end‐tidal sevoflurane concentration of 2%–3% in an air–oxygen mixture, with a fraction of inspired oxygen of 0.5 and a total fresh gas flow of 2 L/min. No additional intravenous anesthesia drugs were administered. Mechanical ventilation was provided using pressure support or pressure‐controlled modes, as appropriate for each patient, with a Datex‐Ohmeda Aestiva/5 MRI anesthesia machine (GE Healthcare, Madison, WI, USA). Upon completion of the MRI procedure, as confirmed by the MRI technologist, the attending anesthesiologist approached the patient in the MRI room and sevoflurane anesthesia was immediately discontinued. In Group P, the attending anesthesiologist administered intravenous propofol at a dose of 0.5 mg/kg, whereas patients in Group N were administered 2 mL of normal saline. Subsequently, patients were transferred to PACU.

Upon arrival at PACU, patients received standard PACU care, including vital sign monitoring, from a PACU nurse. They were observed by an attending anesthesiologist until they met the criteria for awake removal of LMA. The interval from sevoflurane discontinuation immediately after the MRI procedure to meeting the criteria for awake LMA removal—defined as tidal volume more than 5 mL/kg, spontaneous eye opening, and purposeful movement—was recorded as the emergence time. After removal of LMA, patients were observed for EA using the PAED scale every 5 min for 30 min by nurse anesthetist, who was blinded to group allocation and was not involved in the anesthesia care. Three nurse anesthetists, who performed PAED assessment, were trained the use of PAED scale, and the intraclass correlation coefficient was 0.9. EA was defined as a PAED score greater than 12 at any time during the first 30 min of PACU stay. A score threshold of greater than 12 was chosen due to its high sensitivity (100%) and specificity (95%) [22]. The 30‐min assessment time frame was adopted by Mohkamkar et al., who demonstrated that EA predominantly occurs within the first 30 min of PACU stay [23]. If EA occurred, the attending anesthesiologist, who was not blinded to the intervention, was notified to determine whether intravenous propofol at a dose of 1 mg/kg was required as EA rescue medication. The requirement for EA rescue medication was recorded. Parents or legal guardians were allowed to accompany the patients after completion of EA assessment. Any adverse events during PACU stay, including airway obstruction, apnea, laryngospasm, hypotension, bradycardia, and nausea and vomiting, were observed and recorded by PACU nurse, and the attending anesthesiologist was notified for further management. Hypotension was defined as a systolic blood pressure below 70 + (2 × age in years), and bradycardia was defined as heart rate below 60 beats per minute [24]. Patients were discharged from the PACU after meeting the institutional discharge criteria, and the duration of PACU stay was recorded.

### 2.1. Data Collection

Age, sex, body weight, ASA PS, and anesthesia time were recorded as baseline characteristics of the pediatric patients. The primary outcome was the incidence of EA between the two groups. Secondary outcomes included emergence time, duration of PACU stay, adverse events, and requirement for EA rescue medication between the two groups.

### 2.2. Sample Size Calculation

Sample size was calculated based on the hypothesis that intravenous propofol at a dose of 0.5 mg/kg administered at the end of anesthesia would reduce the incidence of EA when compared with the control group. Data from a previous study by Ramlan et al. reported an EA incidence of 25.9% in pediatric patients who received propofol at a dose of 0.5 mg/kg at the end of anesthesia and 51.9% in the control group [10]. Using a two‐sided alpha level of 0.05 and a power of 80%, the minimum required sample size was calculated to be 54 participants per group.

### 2.3. Statistical Analysis

Continuous data were presented as mean and standard deviation (SD) or median and interquartile range (IQR) as appropriate. Categorical data were presented as numbers and percentages. The incidence of EA between the two groups was compared using the chi‐square test. Emergence time and duration of PACU stay, which were not normally distributed as assessed by the Shapiro–Wilk test, were compared using the Mann–Whitney U test. Adverse events and the requirement of EA rescue medication were analyzed using Fisher’s exact test. A risk ratio and median difference with 95% confidence interval were calculated for primary and secondary outcomes, respectively.

The primary analysis was performed using the per‐protocol population, which included patients who completed the study according to the protocol. To assess the robustness of our findings, a sensitivity analysis using the intention‐to‐treat (ITT) principle was additionally performed. The ITT population included all randomized patients (*n* = 130; 65 in each group). Missing primary outcome data for the eight excluded participants were handled using a worst‐case scenario imputation, where all excluded participants in both groups were assumed to have developed EA.

To account for baseline imbalance in ASA PS, a multivariable logistic regression analysis was performed, including the study group and ASA PS as independent variables to determine the independent effect of low‐dose propofol on the incidence of EA. Adjusted odds ratios with 95% confidence intervals were calculated.

A *p* value of less than 0.05 was considered statistically significant. Statistical analysis was performed using IBM SPSS Statistics (Version 29.0.2.0 for Mac; IBM Corp., Armonk, NY, USA).

## 3. Results

**FIGURE 1:**
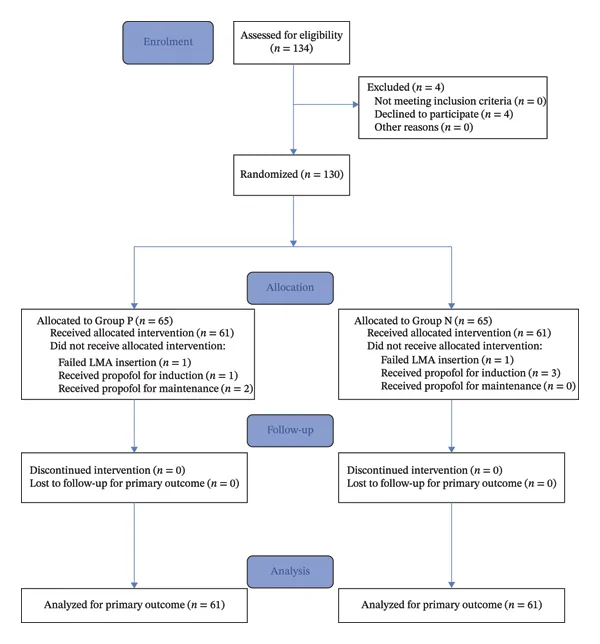
CONSORT flow diagram of the study. Abbreviation: LMA, laryngeal mask airway.

A total of 134 pediatric patients were assessed for eligibility, of whom 4 patients were excluded because their parents declined to participate. Therefore, 130 patients underwent randomization. After randomization, 8 patients (4 in Group P and 4 in Group N) did not receive the allocated intervention due to failed LMA insertion or protocol deviations. Finally, 122 patients were included in the per‐protocol analysis, with 61 patients in each group, as shown in Figure 1.

To confirm the validity of our primary outcome, ITT analysis was additionally performed. The ITT analysis demonstrated a significantly lower incidence of EA in Group P compared with Group N (23.1% vs 43.1%, respectively; *p* = 0.02), with a corresponding risk ratio of 0.54 (95% CI 0.32–0.91). As these findings were consistent with the per‐protocol analysis, the results were primarily presented based on the per‐protocol population.

Baseline characteristics were broadly similar between the two groups, except for ASA PS, which showed a statistically significant difference (*p* = 0.03). All baseline characteristics are summarized in Table 1.

**TABLE 1:** Baseline characteristics of pediatric patients undergoing MRI under sevoflurane anesthesia.

Characteristics	Group P (*n* = 61)	Group N (*n* = 61)	*p* value
Age (years)^a^	5 (3, 6)	5 (3, 6)	0.80
Male^b^	37 (60.7)	32 (52.5)	0.36
Body weight (kg)^a^	16 (13, 20)	18 (15, 21)	0.29
ASA PS^b^			0.03
I	14 (23.0)	25 (41.0)	
II	47 (77.0)	36 (59.0)	
Anesthesia time (minute)^a^	65 (25, 90)	75 (45, 90)	0.51

Abbreviations: ASA PS, American Society of Anesthesiologists physical status; MRI, magnetic resonance imaging.

^a^Data are presented as median with interquartile range, tested by the Mann–Whitney test.

^b^Data are presented as number (percentage), tested by chi‐square test.

**FIGURE 2:**
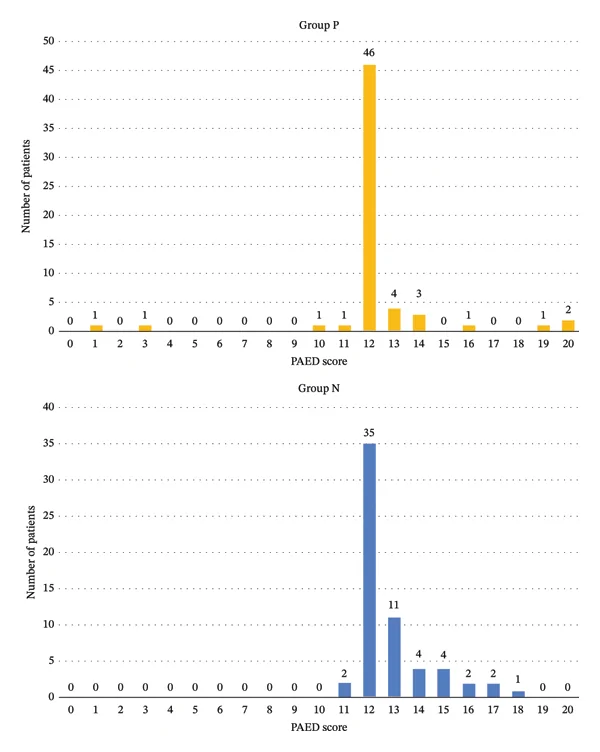
Distribution of peak PAED score of each group. Abbreviation: PAED, pediatric anesthesia emergence delirium.

The incidence of EA was 18.0% in Group P and 39.3% in Group N, showing a statistically significant difference (*p* = 0.01). Patients in Group P had a significantly lower likelihood of developing EA than those in Group N, with a risk ratio of 0.46 (95% CI 0.25–0.85). The distribution of peak PAED score of each group is demonstrated in Figure 2.

After adjusting for ASA PS, Group P remained significantly associated with a lower incidence of EA, with an adjusted odds ratio 0.35 (95% CI 0.15–0.81; *p* = 0.01). ASA PS was not significantly associated with the outcome (*p* = 0.78).

Among patients who developed EA, two patients (3.3%) in Group P required EA rescue medication, compared with one patient (1.6%) in Group N; however, the difference was not statistically significant (*p* = 0.56).

Emergence time and duration of PACU stay did not differ significantly between the two groups, as shown in Table 2. The incidence of adverse events during PACU stay was low in both groups and did not differ significantly (*p* = 0.37). One patient (1.6%) in Group P reported nausea and vomiting, whereas one patient (1.6%) in Group N experienced partial laryngospasm following LMA removal.

**TABLE 2:** The primary and secondary outcomes of the study.

	Group P (*n* = 61)	Group N (*n* = 61)	Estimate of effect (95% CI)	*p* value
Primary outcome				
EA^a^	11 (18.0)	24 (39.3)	RR: 0.46 (0.25–0.85)	0.01^∗^
Secondary outcome				
Emergence time (minutes)^b^	7 (5, 12)	7 (5, 11)	MD: 0.00 (−2.00–2.00)	0.91
Duration of PACU stay (minutes)^b^	75 (70, 85)	80 (70, 85)	MD: 0.00 (−5.00–5.00)	0.93
EA rescue treatment^a^	2 (3.3)	1 (1.6)	RR: 2.00 (0.19–21.41)	1.00
Adverse events^a^			RR: 1.00 (0.06–15.65)	1.00
Laryngospasm	0 (0)	1 (1.6)	—	—
Nausea and vomiting	1 (1.6)	0 (0)	—	—

Abbreviations: EA, emergence agitation; MD, median difference; PACU, postanesthesia care unit; RR, risk ratio.

^∗^Statistically significant as *p* < 0.05.

^a^Data are presented as number (percentage), tested by chi‐square test or Fisher’s exact test if cell expectation count < 5.

^b^Data are presented as median with interquartile range, tested by the Mann–Whitney test.

## 4. Discussion

Aiming to reduce the incidence of EA in pediatric patients undergoing MRI under sevoflurane anesthesia, our randomized controlled trial demonstrated that the incidence of EA was significantly lower in patients receiving intravenous propofol at a dose of 0.5 mg/kg at the end of anesthesia compared with the control group (18.0% vs 39.3%; *p* = 0.01). Our findings showed a lower incidence of EA compared with a previous study by Ramlan et al., which evaluated a similar intervention in pediatric patients undergoing elective surgery and reported EA incidences of 25.9% and 51.9% in the intervention and control groups, respectively [10]. This difference may be explained by procedural characteristics, as painful procedures are more likely to increase the risk of EA than nonpainful procedures.

Our finding also demonstrated that pediatric patients who received intravenous propofol at a dose of 0.5 mg/kg at the end of sevoflurane anesthesia had a significantly lower likelihood of developing EA than those in control group, with a risk ratio of 0.46 (95% CI 0.25–0.85). This result is supported by a previous meta‐analysis study by Xiao et al., which showed that propofol at a dose of 1–3 mg/kg administered at the end of sevoflurane anesthesia reduces the incidence of EA in pediatric patients undergoing elective surgery, with a RR of 0.51 (95% CI 0.39–0.67) [12]. Therefore, low‐dose propofol may effectively reduce EA in pediatric patients who received sevoflurane anesthesia for nonpainful procedures, such as MRI.

From our perspective, EA remains a clinically relevant concern even in pediatric patients undergoing nonpainful procedures. Several identifiable risk factors for EA, including the use of volatile anesthesia and preprocedural anxiety in both patients and parents, are potentially modifiable. In contrast, patient age—particularly between 2 and 6 years, a well‐recognized strong risk factor—cannot be altered. Therefore, perioperative strategies aimed at reducing EA should be proactively implemented, especially in high‐risk pediatric patients. These strategies include age‐appropriate behavioral interventions to minimize preprocedural anxiety (e.g., parental presence at induction and educational cartoon videos), avoidance of volatile anesthesia through the use of total intravenous anesthesia, and perioperative adjunctive pharmacologic agents such as propofol, fentanyl, dexmedetomidine, and ketamine [4, 5, 25].

In addition to propofol, several pharmacologic agents—including fentanyl, dexmedetomidine, and ketamine—have been evaluated for the prevention of EA. A meta‐analysis by Fang et al. demonstrated that these agents significantly reduce EA incidence compared with placebo, reporting odds ratios ranging from 0.17 to 0.28, largely mediated by their sedative properties [26]. Consequently, the selection of a prophylactic agent should be tailored according to its adverse effect profile. High dose of propofol and ketamine may induce respiratory depression or apnea. Opioids like fentanyl increase the risk of postoperative nausea and vomiting, and dexmedetomidine is often associated with hypotension, bradycardia, and delayed emergence [26, 27]. In the present study, no severe adverse events related to propofol—such as hypotension, bradycardia, airway obstruction, or apnea—were observed in Group P, consistent with previous studies [10, 11, 28]. Partial laryngospasm following LMA removal occurred in one patient in Group N during an episode of EA and was successfully resolved with airway maneuvers and positive pressure ventilation. Additionally, one patient in Group P experienced mild postoperative nausea and vomiting that resolved spontaneously without pharmacological intervention required.

Although EA is typically short‐lived and self‐limited, treatment may be required in pediatric patients exhibiting severe symptoms, such as a PAED score greater than 15, prolonged EA, or a risk of self‐injury. After excluding other causes of agitation such as pain or bladder distention, rescue agents—including 0.5–1 mg/kg of propofol, 1–2 mcg/kg of fentanyl, or 0.1 mg/kg of midazolam—may be administered [6]. In our study, most patients who developed EA presented with mild symptoms; only three patients with EA (two in Group P and one in Group N) required EA rescue medication. Per protocol, EA rescue medication was left to the clinical judgment of the attending anesthesiologists. Poststudy interviews revealed that these treatment decisions were primarily prompted by the immediate risk of self‐injury.

Emergence time was not significantly different between the two groups, consistent with the finding reported by Chen et al. [11]. However, a meta‐analysis by Xiao et al. reported that propofol at higher doses of 1–3 mg/kg administered at the end of anesthesia prolonged emergence time, with a mean difference of 4.91 min (95% CI 3.12–6.70), *p* = 0.04 [12]. In our study, median emergence times were comparable between Group P (7 (5, 12)) and Group N (7 (5, 11)), with a median difference of 0.00 (95% CI −2.00–2.00; *p* = 1.00). This demonstrates that a lower dose of propofol at the end of anesthesia may have minimal impact on emergence time.

Several limitations of this study should be acknowledged. First, the trial was conducted at a single center, which may limit the generalizability of the findings. Second, performance bias may have influenced the administration of EA rescue medication, as its requirement was determined by the attending anesthesiologist, who was not blinded to the intervention. Third, the relatively small sample size may have limited our ability to fully evaluate safety outcomes. Fourth, preprocedural anxiety in both patients and parents—a well‐known risk factor for EA—and post‐LMA pharyngeal discomfort were not assessed. Fifth, the long‐term behavioral sequelae of EA were not monitored. Finally, there was a baseline imbalance in ASA PS between the two groups; however, both ASA PS I and II represent low‐risk categories, and ASA PS has not been consistently established as a predictor of EA in the pediatric literature. After adjustment using multivariable logistic regression, ASA PS was not a significant predictor of the outcome, and the treatment effect remained robust.

## 5. Conclusions

In pediatric patients undergoing MRI under sevoflurane anesthesia, administration of intravenous propofol at a dose of 0.5 mg/kg at the end of anesthesia was associated with a significant reduction in the incidence of EA, without evidence of prolonged emergence time or increased adverse events in this study. Further large‐scale, multicenter studies are warranted to confirm the efficacy of this approach and to comprehensively evaluate its safety profile.

## Funding

This study was not supported by any fund and/or organization.

## Ethics Statement

This study was approved by the Ethics Committee of Khon Kaen University for Human Research (HE661348) on August 8, 2023. All participants provided written informed consent prior to enrolment in the study. This research was conducted ethically in accordance with the Declaration of Helsinki.

## Conflicts of Interest

The authors declare no conflicts of interest.

## Data Availability

The data that support the finding of this study are available upon request from the corresponding author. The data are not publicly available due to privacy or ethical restrictions.
